# Dynamic Changes of Metabolic Syndrome Alter the Risks of Cardiovascular Diseases and All-Cause Mortality: Evidence From a Prospective Cohort Study

**DOI:** 10.3389/fcvm.2021.706999

**Published:** 2021-08-04

**Authors:** Di He, Xuhui Zhang, Shuohua Chen, Chen Dai, Qiong Wu, Yaohan Zhou, Ziqi Jin, Shouling Wu, Yimin Zhu

**Affiliations:** ^1^Department of Epidemiology and Biostatistics, School of Public Health, Zhejiang University, Hangzhou, China; ^2^Hangzhou Center for Disease Control and Prevention, Hangzhou, China; ^3^Department of Cardiology, Kailuan General Hospital, Hebei United University, Tangshan, China; ^4^Putuo District People's Hospital, Zhoushan, China; ^5^Department of Respiratory Diseases, Sir Run Run Shaw Hospital Affiliated to School of Medicine, Zhejiang University, Hangzhou, China

**Keywords:** metabolic syndrome, cardiovascular disease, mortality, dynamic changes, onset age

## Abstract

**Background:** Metabolic syndrome (MetS) at baseline increases the risks of cardiovascular diseases (CVD) and all-cause mortality. However, MetS status is changeable during follow-up. The associations of dynamic changes of MetS with CVD and all-cause mortality remain unclear.

**Methods:** Thirty-one thousand four hundred eighty-one eligible subjects were included from the Kailuan cohort. Dynamic changes of MetS were divided into four patterns as MetS-free, MetS-developed, MetS-recovery and MetS-stable. The outcomes were CVD, all-cause mortality, and the subtypes of CVD as myocardial infarction (MI), stroke and heart failure. Multiple Cox regression models were used to calculate the adjusted hazard ratios (HRs) and confidence intervals (95% CIs).

**Results:** Altered risks of CVD, the subtypes of CVD, and all-cause mortality were observed among different dynamic patterns of MetS. Compared with the MetS-free group, MetS-developed group increased the risks of CVD (HR = 1.78, 95% CI = 1.51–2.11), MI (HR = 1.54, 95% CI = 1.01–2.34), stroke (HR = 1.78, 95% CI = 1.45–2.18), and heart failure (HR = 1.63, 95% CI = 1.11–2.39). MetS-recovery group decreased these risks with the HRs of 0.59 (95% CI = 0.48–0.72) for CVD, 0.62 (95% CI = 0.41–0.96) for MI, 0.59 (95% CI = 0.46–0.75) for stroke, and 0.56 (95% CI = 0.34–0.91) for heart failure compared with the MetS-stable group. However, the increased risk in the MetS-developed group and the decreased risk in the MetS-recovery group were not significant for all-cause mortality. When stratified by the onset age of MetS status change, early development of MetS (<50 years) had higher risks of CVD (HR = 2.20, 95% CI = 1.58–3.05), MI (HR = 2.35, 95% CI = 1.00–5.50), stroke (HR = 2.05, 95% CI = 1.38–3.05), heart failure (HR = 2.63, 95% CI = 1.15–6.04), and all-cause mortality (HR = 1.61, 95% CI = 1.13–2.30) than late development (≥50 years). Early recovery of MetS had lower risks with the HRs of 0.38 (95% CI = 0.24–0.59) for CVD, 0.43 (95% CI = 0.18–1.06) for MI, 0.37 (95% CI = 0.21–0.64) for stroke, 0.30 (95% CI = 0.09–1.04) for heart failure, and 0.68 (95% CI = 0.43–1.06) for all-cause mortality than late recovery.

**Conclusion:** Dynamic changes of MetS altered the risks of CVD and all-cause mortality, especially in individuals with an early onset age. These findings highlight the importance of dynamic changes of MetS and onset age on the prevention and control for CVD.

## Introduction

Metabolic syndrome (MetS) is the cluster of metabolic abnormalities as visceral obesity, dyslipidemia, hypertension and hyperglycemia (1). Previous studies have found that MetS increased risks for common chronic diseases such as type 2 diabetes, cardiovascular diseases (CVD), some types of cancers, and all-cause mortality (2–4). MetS and MetS-associated health problems are becoming major social public health issues worldwide (5).

Previous studies mainly examined the associations of MetS status at baseline with the occurrences of adverse long-term outcomes (3, 4, 6, 7). However, during follow-up period, MetS is reversible and changeable depending on the changing lifestyles (8, 9). For example, individuals who are free of MetS at baseline will develop MetS, while those with MetS at baseline will recover from MetS. The baseline MetS status cannot represent overall exposure during follow-up. Therefore, previous cohort studies based on the exposure at baseline cannot correctly evaluate the biological effect of time-varying exposure. The associations between dynamic changes of MetS and risks of adverse long-term outcomes remain to be further elucidated. Park et al. (10) identified four dynamic patterns of MetS, and found that individuals who developed MetS during follow-up had higher risks of major adverse cardiovascular events than those who were consistently free of MetS, while individuals who recovered from MetS decreased these risks compared with the counterparts of stable MetS. However, this study had relatively short follow-up period of 3.5 years and no convincing evidence was found on heart failure and all-cause mortality. Therefore, results with long follow-up period are warranted to comprehensively evaluate the associations of dynamic patterns of MetS with CVD and all-cause mortality.

On the other hand, the changes of MetS status would occur across all age groups. There is no evidence that whether the risks of CVD and all-cause mortality differed with the onset age of MetS change. Theoretically, earlier being MetS means earlier exposure to the organs of cardiovascular system, and that might lead to adverse long-term outcomes. Previous studies also found that early onsets of type 2 diabetes and hypertension were associated with the increased risks of CVD and all-cause mortality (11, 12). In addition, with the rising trend of unhealthy lifestyles in young people, MetS is more frequently diagnosed in adolescents and young adults (13–15). Therefore, identifying the age-specific associations of dynamic patterns of MetS with CVD and all-cause mortality is essential for the efficient management of MetS and precise prevention for CVD.

Using the Kailuan cohort with relatively large sample size and long follow-up period, this study aimed to examine (1) the associations of dynamic changes of MetS with CVD and all-cause mortality, and (2) whether the strengths of these associations differed with the onset age.

## Materials and Methods

### Study Design and Population

The Kailuan cohort is a large prospective study in the Kailuan community of Tangshan City, Hebei province, China. The study design and procedure of this cohort have been described previously in detail (16, 17). Briefly, a total of 101,510 participants aged 18–98 years were recruited from June 2006 to October 2007 at baseline. All participants underwent a comprehensive health examination including epidemiological survey, anthropometric measurement, and biochemical determination. Repeat investigation was carried out biennially. In the current study, we used data of the first three health examinations to define the dynamic patterns of MetS (exposure assessment window). Then, follow-up for long-term outcomes was initiated after the third health examination (follow-up window). Detailed information of this study design was presented in Supplementary Figure 1.

**Figure 1:**
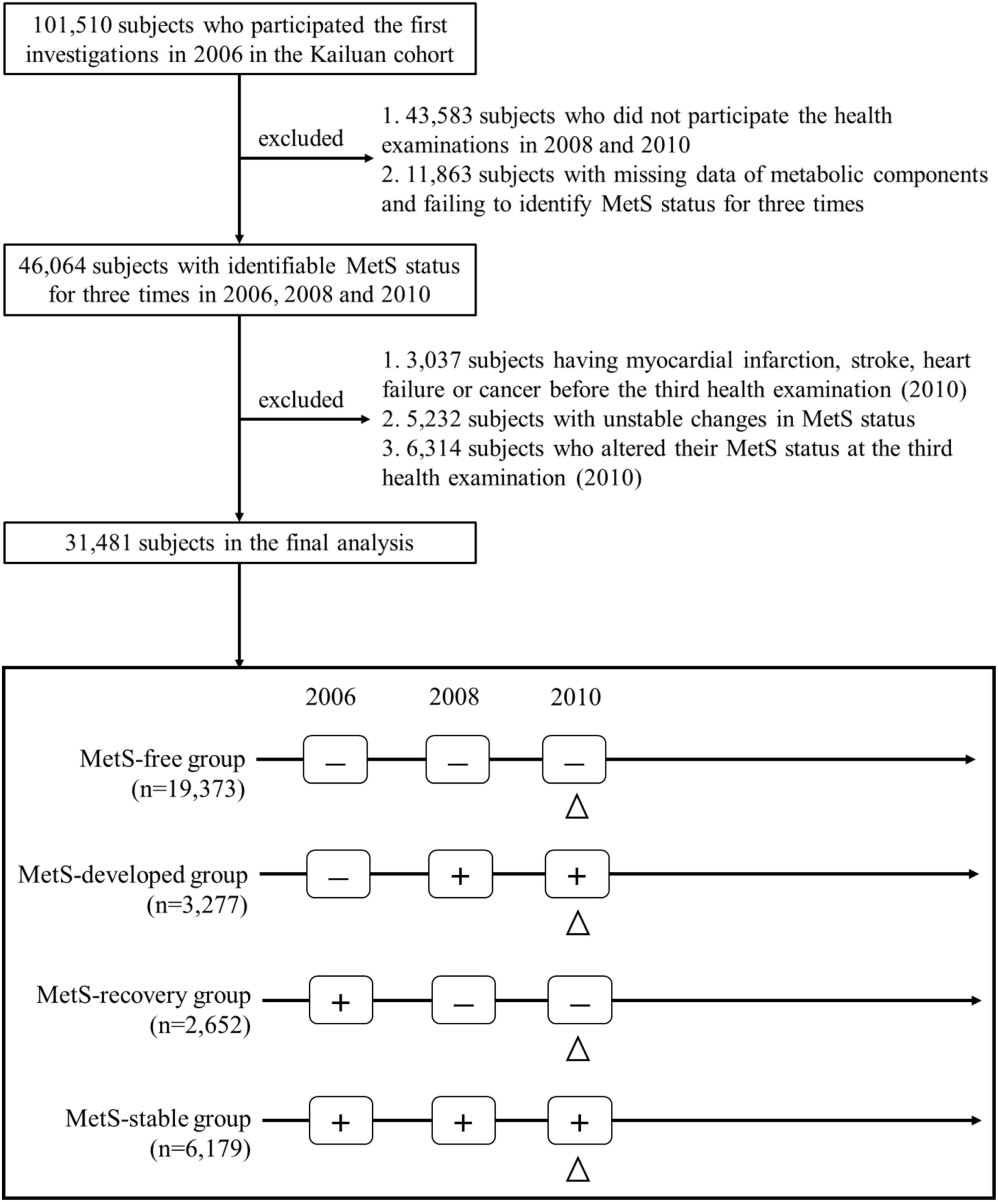
Flowchart of the study population selection. The triangle indicated the initial time of follow-up for long-term outcomes. MetS, metabolic syndrome; “+,” MetS-present status; “-,” MetS-free status.

Figure 1 describes the procedure of the subject selection. This study included subjects with identifiable MetS status for three times in 2006, 2008, and 2010. Among the 46,064 eligible subjects, 14,583 subjects were excluded based on the following exclusion criteria: (1) subjects who had myocardial infarction (MI), stroke, heart failure or cancer before the third health examination; (2) subjects with unstable changes in MetS status; (3) subjects who altered their MetS status at the third health examination. Finally, 31,481 subjects including 19,373 of MetS-free, 3,277 of MetS-developed, 2,652 of MetS-recovery, and 6,179 of MetS-stable, were included for the final analysis. The protocol of this study was approved by the Zhejiang University, School of Medicine and Kailuan General Hospital. All participants provided the written informed consent.

### Data Collection and Assessment

Epidemiological data including socio-demographic information, lifestyles, personal medical history, and family history were collected by the face to face investigation using a standard structured questionnaire. In this study, educational level was categorized as primary school or below, middle, and high school or above. Marital status was categorized as the married and unmarried. Smoking status was divided into two categories as never smokers and ever smokers. Drinking status was categorized as never drinkers and ever drinkers. Ever smokers/drinkers were defined as the subjects who had a history of smoking/drinking or who smoked/drank currently. Physical activity level was evaluated based on the frequency of moderate and vigorous physical activities, and inactive physical activity was defined as <1 time per week. Total salt intake was classified as ≤ 10 g/day and >10 g/day (high salt intake).

Anthropometric data including height, weight, waist circumference (WC), and blood pressure were measured by trained investigators following standard procedures as previously described (16, 17). Height and weight were measured with the subjects wearing no shoes and light clothing. Body mass index (BMI) was calculated as weight (kg) divided by the square of height (m^2^). WC was measured at the midpoint of the lowest rib and iliac crest. Blood pressure was measured with a mercury sphygmomanometer in a seated position. Systolic blood pressure (SBP) and diastolic blood pressure (DBP) were calculated as the average of three measurements at 30-s intervals. Blood samples were collected from each subject after an overnight fast. Biochemical indicators, including fasting blood glucose (FBG), triglyceride (TG), total cholesterol (TC), low-density lipoprotein cholesterol (LDL-C), and high-density lipoprotein cholesterol (HDL-C), were measured using an auto-analyzer (Hitachi 747, Tokyo, Japan). FBG was analyzed with the hexokinase/glucose-6-phosphate dehydrogenase method. TG, TC, LDL-C, and HDL-C were measured using the enzymatic colorimetric method.

### Definition of MetS and Dynamic Patterns of MetS

MetS was defined by the widely used harmonizing criteria (18), and subjects presenting three or more metabolic abnormalities were diagnosed as MetS. Metabolic abnormalities included: (1) elevated WC (≥85 cm for men, ≥80 cm for women in China); (2) elevated TG (≥1.7 mmol/L) or using lipid-lowering drugs; (3) reduced HDL-C (<1.03 mmol/L for men, <1.29 mmol/L for women) or using lipid-lowering drugs; (4) elevated blood pressure (SBP ≥130 mmHg and/or DBP ≥85 mmHg) or using antihypertensive drugs; (5) elevated FBG (≥5.6 mmol/L) or using antidiabetic drugs. According to the MetS status in the first three health examinations, four dynamic patterns of MetS were included in the current analysis as described in Figure 1. MetS-free was defined as the subjects who were consistently free of MetS in 2006, 2008, and 2010. MetS-stable was those who presented MetS throughout three health examinations. MetS-developed was defined as the subjects who were free of MetS in 2006, but developed MetS in the following two health examinations, and MetS-recovery was those who presented MetS in 2006, but became free of MetS in the following two health examinations. In addition, two dynamic patterns of MetS were excluded in the current analysis as described in Supplementary Figure 2. Unstable change of MetS was defined as the subjects who presented no consistent change of MetS status, and change at the third examination was those who altered their MetS status at the third examination. Age of the MetS development and recovery was defined as the age at which MetS status first changed (i.e., the age of subjects in the second health examinations). Using the cut-value of 50 years, we defined early development and recovery of MetS as the subjects whose age of MetS development and recovery <50 years. In contrast, late development and recovery were defined as those whose age ≥50 years.

### Outcomes Ascertainment and Follow-Up

The primary outcomes in this study were CVD and all-cause mortality. CVD was defined as the composite of MI, stroke, and heart failure. The outcome data of MI, stroke and HF were obtained from hospital charge register and local Municipal Social Insurance Institution. We used the ICD-10th edition codes to identify the incident cases. An expert panel collected and reviewed the diagnostic records of MI, stroke and HF from 11 local hospitals to ensure the accuracy of the outcome data. The detailed diagnostic criteria were as follows: MI was diagnosed based on the electrocardiogram, chest pain symptoms, and dynamic changes of cardiac enzyme (19). Stroke was determined by the World Health Organization criteria (20) combining with brain computed tomography or MRI for confirmation, and was further divided into two subtypes as ischemic stroke and hemorrhagic stroke. Heart failure was diagnosed by the clinical symptoms, echocardiography, electrocardiogram, and chest X-ray, following the criteria of the European Society of Cardiology (21). All-cause mortality data were collected by death certificates from provincial vital statistics offices. In the current study, follow-up was initiated after the third health examination (2010) and ended on the time of the first occurrence of the outcome, death or censoring date, whichever came first. The censoring dates were December 31, 2017 for CVD, MI and stroke, December 31, 2016 for heart failure, and December 31, 2019 for all-cause mortality.

### Statistical Analysis

Continuous variables with normal distribution were presented as mean (standard deviation), while continuous variables with skewed distribution were presented as median (interquartile range). Categorical variables were presented as number (percentage). Comparisons of the characteristics among the four dynamic patterns of MetS were performed using one-way ANOVA for continuous variables, and chi-square test or Fisher exact test for categorical variables.

To analyze the associations of dynamic patterns of MetS with CVD and all-cause mortality, multiple Cox regression models were used to calculate the adjusted hazard ratios (HRs) and confidence intervals (95% CIs) for MetS-developed, MetS-recovery, and MetS-stable groups using MetS-free group as reference. The covariates included age, sex, marital status, education level, smoking status, drinking status, physical activity level, salt intake, use of anti-hypertensive drug, anti-diabetic drug, lipid-lowering drug, and family history of myocardial infarction, stroke. To evaluate the effect of MetS development, we performed the comparison between MetS-free and MetS-developed groups using MetS-free group as reference. Similarly, to evaluate the effect of MetS recovery, we performed the comparison between MetS-recovery and MetS stable groups using MetS-stable group as reference.

To investigate the associations of MetS development and recovery age with CVD and all-cause mortality, we performed stratified analyses by the age of MetS development and recovery (<50 years and ≥50 years), then explored the interaction between the age groups and the dynamic patterns of MetS.

To examine the robustness of our results, we conducted the sensitivity analyses after excluding incident events during the first 2 years of follow-up to minimize reverse causality.

All the analyses were carried out using R software (Version 4.0.2). All the *P*-values were two-sided, and *P* < 0.05 was considered as statistically significant.

## Results

### Baseline Characteristics of the Subjects

Among the 31,481 eligible subjects at baseline, the mean age was 52.4 years and 22.7% were female. The average follow-up period were 7.1 years for CVD and 9.1 years for all-cause mortality. Baseline characteristics of the subjects by the dynamic patterns of MetS are summarized in Table 1. MetS-stable group consisted of the oldest subjects, then followed by MetS-recovery and MetS-developed groups. MetS-stable group also had the highest percentages of hypertension (76.8%), diabetes (36.8%), hyperlipidemia (13.7%), and family histories of MI (2.62%), stroke (5.32%), the highest means of BMI (27.5 ± 3.1 kg/m^2^), SBP (143.5 ± 18.4 mmHg), DBP (90.4 ± 10.7 mmHg), WC (95.2 ± 8.9 cm), FPG (6.94 ± 2.63 mmol/L), TG (2.85 ± 2.53 mmol/L), and the lowest mean of HDL-C (1.38 ± 0.42 mmol/L). In addition, both MetS-stable and MetS-developed groups had higher percentages of ever smokers (40.3 and 43.4%), ever drinkers (37.3 and 40.8%), and high salt intake (12.4 and 12.4%), while these unhealthy lifestyles were less common in MetS-free and MetS-recovery groups. The characteristics of the subjects in 2006 and 2008 are summarized in Supplementary Tables 1, 2. Based on the information of the three tables (Table 1 and Supplementary Tables 1, 2), several changes of the characteristics were found from 2006 to 2010. For lifestyles, the percentage of ever drinkers decreased, while the percentage of inactive physical activity increased among the four dynamic patterns from 2006 to 2010. There was no significant change in the distributions of ever smokers and high salt intake during the 4 years. For metabolic components, the means of SBP, DBP, WC, FBG, TG increased and HDL-C decreased in the MetS-developed group from 2006 to 2010. In contrast, decreased means of SBP, DBP, WC, FBG, and TG were observed in the MetS-recovery group during the 4 years.

**Table 1:** Baseline characteristics of subjects by the dynamic patterns of MetS in 2010.

	**MetS-free**	**MetS-developed**	**MetS-recovery**	**MetS-stable**	***P*-value**
Number	19,373	3,277	2,652	6,179	
Mean age (SD), year	50.8 (12.2)	53.7 (11.2)	54.0 (11.4)	56.0 (10.5)	<0.001
Female, n (%)	4,733 (24.4)	627 (19.1)	483 (18.2)	1,291 (20.9)	<0.001
Mean BMI (SD), kg/m^2^	23.7 (3.0)	26.7 (3.1)	25.3 (3.2)	27.5 (3.1)	<0.001
High school or above, n (%)	5,376 (27.8)	680 (20.8)	530 (20.0)	1,164 (18.8)	<0.001
Married, n (%)	19,071 (98.4)	3,248 (99.1)	2,619 (98.8)	6,119 (99.0)	<0.001
Ever smokers, n (%)	7,253 (37.4)	1,421 (43.4)	944 (35.6)	2,491 (40.3)	<0.001
Ever drinkers, n (%)	6,404 (33.1)	1,338 (40.8)	839 (31.6)	2,303 (37.3)	<0.001
Inactive physical activity, n (%)	6,169 (31.8)	1,073 (32.7)	718 (27.1)	1,860 (30.1)	<0.001
High salt intake, n (%)	1,872 (9.7)	405 (12.4)	236 (8.9)	766 (12.4)	<0.001
Family history of MI, n (%)	368 (1.90)	74 (2.26)	45 (1.70)	162 (2.62)	0.002
Family history of stroke, n (%)	834 (4.30)	156 (4.76)	102 (3.85)	329 (5.32)	0.002
Mean SBP (SD), mmHg	123.5 (17.1)	138.9 (17.0)	131.3 (18.5)	143.5 (18.4)	<0.001
Mean DBP (SD), mmHg	80.6 (9.8)	89.2 (10.1)	84.8 (10.3)	90.4 (10.7)	<0.001
Mean WC (SD), cm	83.9 (9.7)	93.6 (8.5)	88.2 (9.6)	95.2 (8.9)	<0.001
Mean FBG (SD), mmol/L	5.15 (0.98)	6.14 (1.59)	5.35 (1.21)	6.94 (2.63)	<0.001
Mean TG (SD), mmol/L	1.17 (0.79)	2.47 (2.17)	1.37 (0.85)	2.85 (2.53)	<0.001
Mean HDL-C (SD), mmol/L	1.61 (0.47)	1.44 (0.53)	1.51 (0.38)	1.38 (0.42)	<0.001
MetS components, n (%)					<0.001
0	3,802 (19.6)	0 (0)	145 (5.5)	0 (0)	
1	7,629 (39.4)	0 (0)	733 (27.6)	0 (0)	
2	7942 (41.0)	0 (0)	1,774 (66.9)	0 (0)	
3	0 (0)	2,165 (66.1)	0 (0)	3,116 (50.4)	
4	0 (0)	967 (29.5)	0 (0)	2,421 (39.2)	
5	0 (0)	145 (4.4)	0 (0)	642 (10.4)	
Elevated WC, n (%)^a^	9,697 (50.1)	3,124 (95.3)	1,784 (67.3)	5,899 (95.5)	<0.001
Hypertension, n (%)^b^	5,172 (26.7)	2,194 (66.9)	1,176 (44.3)	4,745 (76.8)	<0.001
Diabetes, n (%)^c^	387 (2.0)	555 (16.9)	153 (5.8)	2,275 (36.8)	<0.001
Hyperlipidemia, n (%)	348 (1.8)	263 (8.0)	87 (3.3)	849 (13.7)	<0.001
Anti-hypertensive drug use, n (%)	702 (3.62)	560 (17.09)	222 (8.37)	1,633 (26.43)	<0.001
Anti-diabetic drug use, n (%)	79 (0.41)	128 (3.91)	49 (1.85)	764 (12.36)	<0.001
Lipid-lowering drug use, n (%)	11 (0.06)	50 (1.53)	1 (0.04)	194 (3.14)	<0.001

a *Elevated WC was determined by the measured WC ≥85 cm for men or ≥80 cm for women*.

b *Hypertension was determined by the measured blood pressure ≥140/90 mmHg or use of the anti-hypertensive drug*.

c *Diabetes was determined by the measured FBG ≥7.0 mmol/L or use of the anti-diabetic drug*.

*MetS, metabolic syndrome; BMI, body mass index; MI, myocardial infarction; SBP, systolic blood pressure; DBP, diastolic blood pressure; WC, waist circumference; FBG, fasting blood glucose; TG, triglyceride; HDL-C, high-density lipoprotein cholesterol; SD, standard deviation*.

### Associations of the Dynamic Patterns of MetS With CVD and All-Cause Mortality

**Figure 2:**
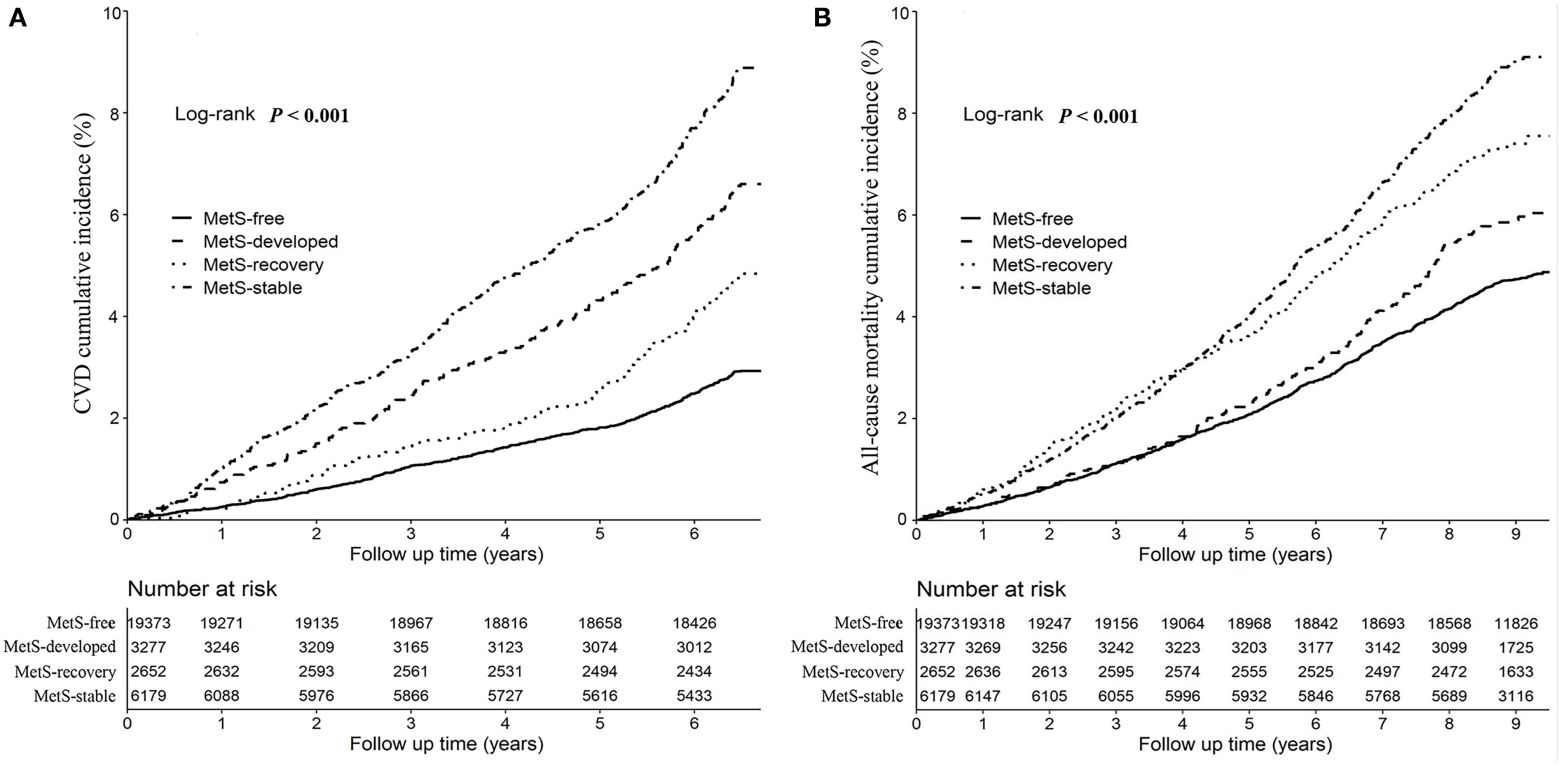
Kaplan-Meier cumulative incidence curves of cardiovascular disease **(A)** and all-cause mortality **(B)** by the dynamic patterns of MetS. Cardiovascular disease was the composite of myocardial infarction, stroke, and heart failure. CVD, cardiovascular disease; MetS, metabolic syndrome.

**Figure 3:**
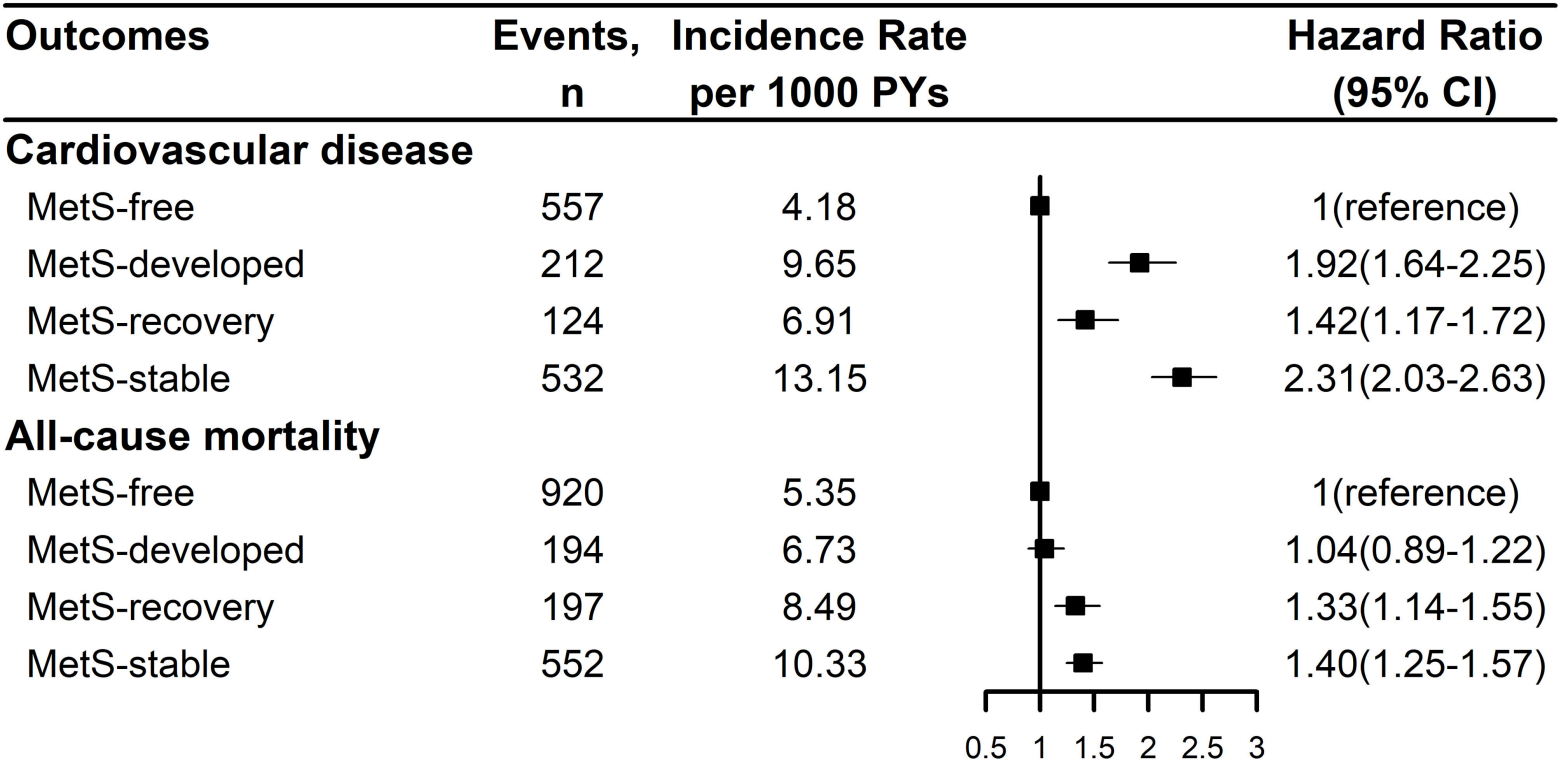
Incidence rates and risks of cardiovascular disease and all-cause mortality by the dynamic patterns of MetS. Cardiovascular disease was the composite of myocardial infarction, stroke, and heart failure. Hazard ration was adjusted for age, sex, marital status, education level, smoking status, drinking status, physical activity level, salt intake, use of anti-hypertensive drug, anti-diabetic drug, lipid-lowering drug, and family history of myocardial infarction, stroke. MetS, metabolic syndrome; PY, person-year; CI, confidence interval.

**Figure 4:**
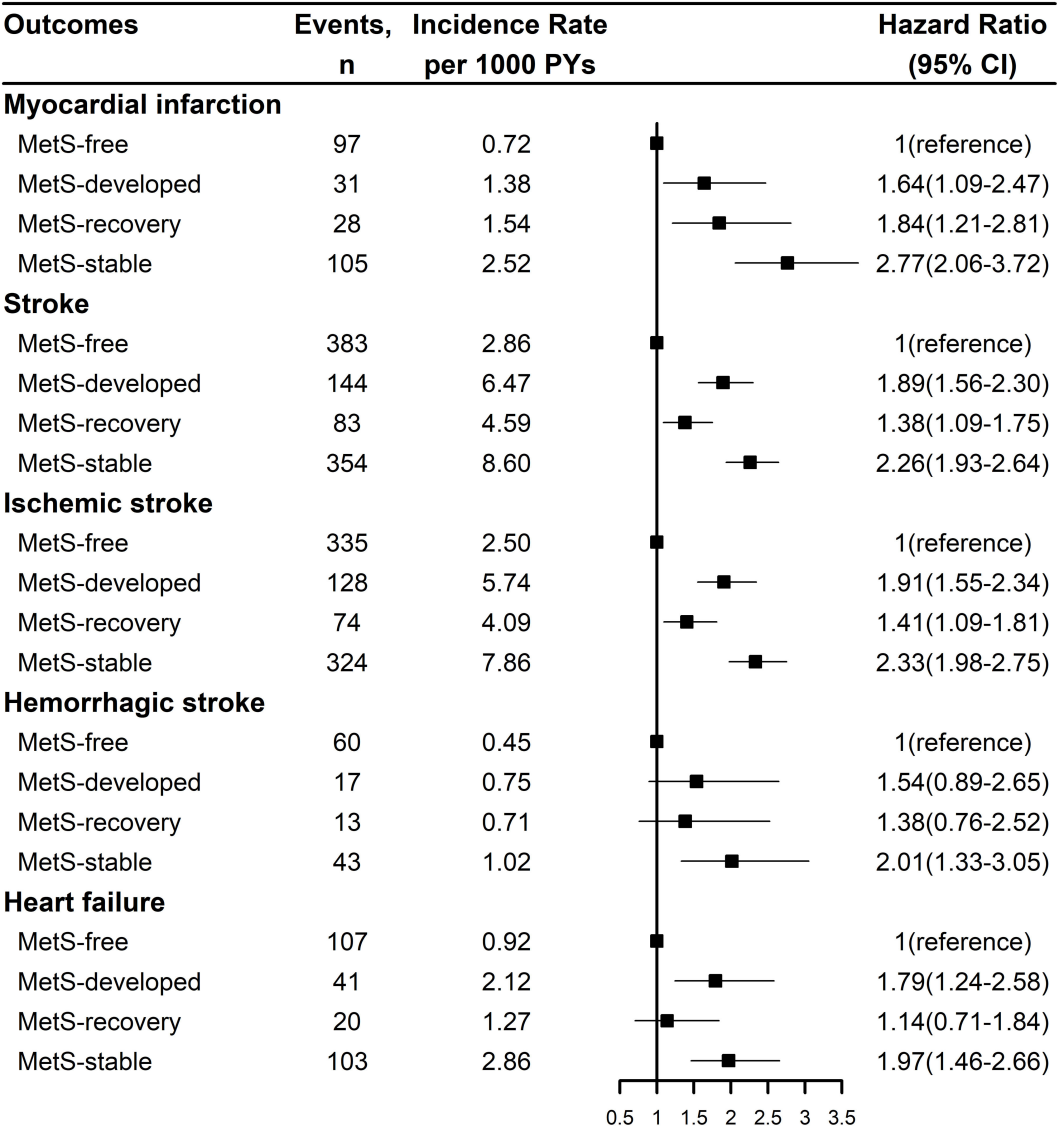
Incidence rates and risks of the subtypes of cardiovascular disease by the dynamic patterns of MetS. Stroke was the composite of ischemic stroke and hemorrhagic stroke. Hazard ration was adjusted for age, sex, marital status, education level, smoking status, drinking status, physical activity level, salt intake, use of anti-hypertensive drug, anti-diabetic drug, lipid-lowering drug, and family history of myocardial infarction, stroke. MetS, metabolic syndrome; PY, person-year; CI, confidence interval.

Figure 2 showed the Kaplan-Meier cumulative incidence curves of CVD and all-cause mortality by the dynamic patterns of MetS. Different cumulative incidence curves were found for CVD and all-cause mortality among different dynamic patterns of MetS (Log-rank *P* < 0.001). For CVD, MetS-stable group had the highest cumulative incidence throughout the follow-up period, then followed by MetS-developed and MetS-recovery groups, while MetS-free group had the lowest cumulative incidence. For all-cause mortality, MetS-stable and MetS-recovery groups had the highest cumulative incidence in the first 4.5 years of follow-up, but MetS-stable group had higher cumulative incidence in the later follow-up. In contrast, MetS-developed and MetS-free groups had the lowest cumulative incidence in the first 4 years of follow-up, but MetS-developed group had higher cumulative incidence in the later follow-up. Figure 3 describes the incidence rates and risks of CVD and all-cause mortality by the dynamic MetS patterns. MetS-stable group had the highest incidence rates of 13.15/1,000 person years for CVD and 10.33/1,000 person years for all-cause mortality, while MetS-free group had the lowest incidence rates for these two outcomes. For CVD, MetS-developed group had higher incidence rate than MetS-recovery group, but this trend was inverse for all-cause mortality. After controlling for the confounding factors, MetS-stable group had the highest risks of CVD (HR = 2.31, 95% CI = 2.03–2.63) and all-cause mortality (HR = 1.40, 95% CI = 1.25–1.57) compared with the MetS-free group. For the subtypes of CVD, similar results were found with CVD (Figure 4). MetS-stable group had the highest incidence rates and risks of MI, stroke and heart failure, then followed by MetS-developed and MetS-recovery groups, while MetS-free group had the lowest incidence rates and risks. In addition, for the subtypes of stroke, ischemic stroke seemed to have a stronger association with the dynamic patterns of MetS than hemorrhagic stroke.

### Associations of the MetS Development and Recovery With CVD and All-Cause Mortality

The overall effects of MetS development and recovery on CVD and all-cause mortality are summarized in Table 2. Compared with the MetS-free group, MetS-developed group increased the risk of CVD with the HR of 1.78 (95% CI = 1.51–2.11), and MetS-recovery group decreased the risk of CVD with the HR of 0.59 (95% CI = 0.48–0.72) compared with the MetS-stable group. However, the increased risk in the MetS-developed group and the decreased risk in the MetS-recovery group were not significant for all-cause mortality. For the subtypes of CVD, MetS-developed group increased the risks of MI (HR = 1.54, 95% CI = 1.01–2.34), stroke (HR = 1.78, 95% CI = 1.45–2.18), and heart failure (HR = 1.63, 95% CI = 1.11–2.39) compared with the MetS-free group (Table 3). In contrast, when compared with the MetS-stable group, MetS-recovery group decreased these risks with the HRs of 0.62 (95% CI = 0.41–0.96) for MI, 0.59 (95% CI = 0.46–0.75) for stroke, and 0.56 (95% CI = 0.34–0.91) for heart failure (Table 4). When stratified by the subtypes of stroke, the risk of ischemic stroke was significantly increased in the MetS-developed group (HR = 1.79, 95% CI = 1.44-2.22) and decreased in the MetS-recovery group (HR = 0.58, 95% CI = 0.45–0.76), but that association was not significant for hemorrhagic stroke.

**Table 2:** Hazard ratios and 95%CIs of the cardiovascular disease and all-cause mortality in MetS-developed and MetS-recovery groups according to the age of MetS status change.

**Outcomes**	**Hazard ratio (95% CI)** ^****†****^			***P*_**interaction**_**
	**All age**	** <50 years**	**≥50 years**	
**MetS-developed vs. MetS-free**				
Cardiovascular disease				
MetS-free	1 (reference)	1 (reference)	1 (reference)	0.003
MetS-developed	1.78 (1.51–2.11)	2.20 (1.58–3.05)	1.64 (1.35–1.99)	
All-cause mortality				
MetS-free	1 (reference)	1 (reference)	1 (reference)	<0.001
MetS-developed	1.03 (0.87–1.21)	1.61 (1.13–2.30)	0.93 (0.77–1.11)	
**MetS-recovery vs. MetS-stable**				
Cardiovascular disease				
MetS-stable	1 (reference)	1 (reference)	1 (reference)	0.005
MetS-recovery	0.59 (0.48–0.72)	0.38 (0.24–0.59)	0.67 (0.54–0.85)	
All-cause mortality				
MetS-stable	1 (reference)	1 (reference)	1 (reference)	0.030
MetS-recovery	0.95 (0.80–1.12)	0.68 (0.43–1.06)	1.00 (0.84–1.21)	

*Cardiovascular disease was the composite of myocardial infarction, stroke, and heart failure*.

† *Hazard ration was adjusted for age, sex, marital status, education level, smoking status, drinking status, physical activity level, salt intake, use of anti-hypertensive drug, anti-diabetic drug, lipid-lowering drug, and family history of myocardial infarction, stroke*.

*MetS, metabolic syndrome; CI, confidence interval*.

**Table 3:** Hazard ratios and 95%CIs of the subtypes of cardiovascular disease in the MetS-developed group according to the age of MetS development.

**Outcomes**	**Hazard ratio (95% CI)** ^****†****^			***P*_**interaction**_**
	**All age**	** <50 years**	**≥50 years**	
**Myocardial infarction**				
MetS-free	1 (reference)	1 (reference)	1 (reference)	0.080
MetS-developed	1.54 (1.01–2.34)	2.35 (1.00–5.50)	1.35 (0.83–2.20)	
**Stroke**				
MetS-free	1 (reference)	1 (reference)	1 (reference)	0.047
MetS-developed	1.78 (1.45–2.18)	2.05 (1.38–3.05)	1.66 (1.31–2.10)	
**Ischemic stroke**				
MetS-free	1 (reference)	1 (reference)	1 (reference)	0.055
MetS-developed	1.79 (1.44–2.22)	2.04 (1.32–3.15)	1.68 (1.31–2.15)	
**Hemorrhagic stroke**				
MetS-free	1 (reference)	1 (reference)	1 (reference)	0.675
MetS-developed	1.42 (0.81–2.48)	1.53 (0.60–3.92)	1.39 (0.69–2.80)	
**Heart failure**				
MetS-free	1 (reference)	1 (reference)	1 (reference)	0.055
MetS-developed	1.63 (1.11–2.39)	2.63 (1.15–6.04)	1.44 (0.93–2.24)	

*Stroke was the composite of ischemic stroke and hemorrhagic stroke*.

† *Hazard ration was adjusted for age, sex, marital status, education level, smoking status, drinking status, physical activity level, salt intake, use of anti-hypertensive drug, anti-diabetic drug, lipid-lowering drug, and family history of myocardial infarction, stroke*.

*MetS, metabolic syndrome; CI, confidence interval*.

**Table 4:** Hazard ratios and 95%CIs of the subtypes of cardiovascular disease in the MetS- recovery group according to the age of MetS recovery.

**Outcomes**	**Hazard ratio (95% CI)** ^****†****^			***P*_**interaction**_**
	**All age**	** <50 years**	**≥50 years**	
**Myocardial infarction**				
MetS-stable	1 (reference)	1 (reference)	1 (reference)	0.253
MetS-recovery	0.62 (0.41–0.96)	0.43 (0.18–1.06)	0.73 (0.45–1.20)	
**Stroke**				
MetS-stable	1 (reference)	1 (reference)	1 (reference)	0.013
MetS-recovery	0.59 (0.46–0.75)	0.37 (0.21–0.64)	0.69 (0.52–0.90)	
**Ischemic stroke**				
MetS-stable	1 (reference)	1 (reference)	1 (reference)	0.032
MetS-recovery	0.58 (0.45–0.76)	0.39 (0.22–0.70)	0.66 (0.49–0.89)	
**Hemorrhagic stroke**				
MetS-stable	1 (reference)	1 (reference)	1 (reference)	0.129
MetS-recovery	0.64 (0.34–1.21)	0.18 (0.02–1.42)	0.83 (0.42–1.64)	
**Heart failure**				
MetS-stable	1 (reference)	1 (reference)	1 (reference)	0.179
MetS-recovery	0.56 (0.34–0.91)	0.30 (0.09–1.04)	0.63 (0.36–1.08)	

*Stroke was the composite of ischemic stroke and hemorrhagic stroke*.

† *Hazard ration was adjusted for age, sex, marital status, education level, smoking status, drinking status, physical activity level, salt intake, use of anti-hypertensive drug, anti-diabetic drug, lipid-lowering drug, and family history of myocardial infarction, stroke*.

*MetS, metabolic syndrome; CI, confidence interval*.

### Associations of the MetS Development Age With CVD and All-Cause Mortality

When stratified by the age (<50 years and ≥50 years) of MetS development, risk differences of CVD and all-cause mortality were found between different age groups (Table 2). Compared with the MetS-free group in the same age group, individuals with early development of MetS (<50 years) had higher risks of CVD (HR = 2.20, 95% CI = 1.58–3.05) and all-cause mortality (HR = 1.61, 95% CI = 1.13–2.30) than those with late development. Significant interactions between MetS development and development age were found for CVD (*P*_interaction_ = 0.003) and all-cause mortality (*P*_interaction_ < 0.001). For the subtypes of CVD, individuals with early development of MetS also had higher risks with the HRs of 2.35 (95% CI = 1.00–5.50) for MI, 2.05 (95% CI = 1.38–3.05) for stroke, and 2.63 (95% CI = 1.15–6.04) for heart failure than those with late development (Table 3). Significant interactions were found for stroke (*P*_interaction_ = 0.047), and marginal significances for MI (*P*_interaction_ = 0.080) and heart failure (*P*_interaction_ = 0.055). In addition, for the subtypes of stroke, the significant risk difference between early and late development of MetS was observed for ischemic stroke, but that was not obvious for hemorrhagic stroke (Table 3).

### Associations of the MetS Recovery Age With CVD and All-Cause Mortality

Compared with the MetS-stable group in the same age group, individuals with early recovery of MetS (<50 years) had lower risks of CVD (HR = 0.38, 95% CI = 0.24–0.59) and all-cause mortality (HR = 0.68, 95% CI = 0.43–1.06) than those with late recovery (Table 2). Significant interactions between MetS recovery and recovery age were found for CVD (*P*_interaction_ = 0.005) and all-cause mortality (*P*_interaction_ = 0.030). For the subtypes of CVD, individuals with early recovery of MetS also had lower risks with the HRs of 0.43 (95% CI = 0.18–1.06) for MI, 0.37 (95% CI = 0.21–0.64) for stroke, and 0.30 (95% CI = 0.09–1.04) for heart failure than those with late recovery (Table 4). Significant interaction was found for stroke (*P*_interaction_ = 0.013). Similarly, the risk difference between early and late recovery of MetS was significant for ischemic stroke, but not for hemorrhagic stroke (Table 4).

### Sensitivity Analyses

After excluding the incident events during the first 2 years of follow-up, consistent results were found with the original analyses. Different incidence rates and risks of CVD, all-cause mortality, and the subtypes of CVD were observed among different dynamic patterns of MetS (Supplementary Figures 3, 4). When stratified by the age of MetS development and recovery, early development of MetS still had higher risks of CVD, all-cause mortality, and the subtypes of CVD than late development, while early recovery of MetS had lower risks than early recovery (Supplementary Tables 3–5).

## Discussion

In this prospective cohort study, we found that dynamic changes of MetS altered the risks of CVD, the subtypes of CVD, and all-cause mortality. Individuals who developed MetS increased these risks than MetS-free counterparts, while those who recovered from MetS decreased these risks compared with MetS-stable individuals. Furthermore, higher risks of CVD, the subtypes of CVD, and all-cause mortality were found in individuals with early development of MetS than those with late development, and lower risks were observed in individuals with early recovery of MetS compared with the late recovery counterparts.

Previous studies had found that MetS increased the risks of CVD and all-cause mortality (3, 4, 6, 7). These findings were mainly based on the MetS status at baseline. However, due to the reversible and changeable status of MetS during follow-up, we should make clear that how these dynamic changes of MetS affected the long-term outcomes. Park et al. (10) found that dynamic changes of MetS altered the risks of major adverse cardiovascular events. The present results were consistent with the previous finding on MI and stroke. Furthermore, our results extended the associations on heart failure and all-cause mortality.

Different MetS dynamic patterns had different risks of CVD and all-cause mortality. Development of MetS increased the risks of CVD and its subtypes compared with MetS-free counterparts, which reflected the significant harm associated with the MetS development. On the other hand, our results showed decreased risks of CVD and its subtypes for recovery from MetS than stable MetS. Koskinen et al. (22) found that recovery from MetS had smaller intima-media thickness, higher carotid artery distensibility, and higher flow-mediated dilatation. The recovery group could reduce intima-media thickness progression and carotid artery distensibility change, which might decrease the risk of CVD. These results were also supported by the facts that lifestyle interventions on MetS would reduce the incidences of consequent long-term outcomes (23, 24). However, our results indicated that the dynamic changes of MetS mainly affected the risks of ischemic disease, while the biological effects for hemorrhagic disease and all-cause mortality were not significant. This might attribute to the different pathological mechanisms. Ischemic disease was mainly caused by the atherosclerosis, and MetS was considered as an important risk factor for developing atherosclerosis (4, 6). MetS could cause the accumulation of lipids and systemic inflammation, which accelerated the process of atherosclerosis, and then led to the ischemic disease. In addition, it was worth noting that the risks of all-cause mortality and MI in the MetS-recovery group were higher than those in the MetS-developed group. The reason for the abnormal result might be the exposure duration bias. MetS-recovery was defined as the subjects who had MetS in 2006, and then recovered into MetS-free in 2008 and 2010. The subjects with MetS in 2006 might have long exposure to MetS before 2006, and this would overestimate the risks in the MetS-recovery group.

Besides the overall MetS dynamic patterns, our study found that the age of MetS status change was associated with the risks of CVD and all-cause mortality. Early development of MetS had higher risks than late development. This age-specific association was consistent with the previous findings on hypertension (12), type 2 diabetes (11), and LDL-C (25). One potential mechanism might be that the individuals with early development of MetS had higher percentages of unhealthy lifestyles, leading to the higher levels of obesity, metabolic abnormalities, and consequent long-term outcomes. On the other hand, Niiranen et al. (26) found that early-onset hypertension was more affected by genetic susceptibility. Similar result was also reported on the early-onset type 2 diabetes (27). Furthermore, numerous genetic variants were found to be associated with both MetS and CVD (28–30). Therefore, another plausible explanation could be raised that early development of MetS was more likely to be genetically susceptible. Individuals with early-onset MetS might carry more MetS-related genetic variants, which would result in the excess risks of CVD. Encouragingly, despite the higher risks for early-onset MetS, lower risks of CVD and all-cause mortality were observed in the individuals with early recovery of MetS. Based on this evidence, we could infer that early interventions for MetS would efficiently prevent the CVD and mortality.

These findings have some implications in clinical and public health practices. First, altered risks of CVD and all-cause mortality in different MetS dynamic patterns emphasize the need to promote regular screening for MetS. Individuals with developed or stable MetS should be considered as the targeted population for control and prevention. Furthermore, higher risks in individuals with early development of MetS suggest the importance of focusing on the cardiometabolic health among young adults. Although the incidence of MetS is relatively low among young adults (13–15), most of the young MetS patients are unaware of their elevated cardiovascular and mortality risks, which in turn, leads to great disease burden in the future. Therefore, comprehensive interventions on lifestyles and stringent clinical treatments should be performed as early as possible for individuals with developed or stable MetS. On the other hand, our results found decreased risks among the individuals who recovered from MetS, especially for young adults. This finding confirms the great benefits of taking effective interventions as early as possible. Our results also highlight the necessity to include the dynamic changes of MetS and onset age in the traditional MetS guidelines.

There are several strengths in this study including the prospective cohort design, large sample size, and standardized evaluation of the metabolic markers and body sizes. To our knowledge, this study is the first to investigate the associations of dynamic changes of MetS with heart failure and all-cause mortality, and the effects of development and recovery age on the long-term outcomes. Additionally, we focused on the incident MetS changes that occurred during follow-up. Therefore, the potential prevalence-incidence bias would be decreased. This study also has some limitations. First, the subjects were mainly occupational workers from Kailuan community in Northern China, and 77.3% of the subjects were males. This might restrict the generalization of the findings. However, previous study found that the biological effect of MetS on CVD was little difference between males and females (31). Second, the dynamic changes of MetS were assessed with three consecutive investigations in 2006, 2008 and 2010. More times of investigations would more precisely evaluate the dynamic patterns. However, due to the restriction on the follow-up period and sample size, more accurate dynamic patterns of MetS could not be constructed in the current study. Comprehensive evaluations for the dynamic patterns of MetS would be desired in the future. Third, subjects with MetS in 2006 might have long exposure to MetS before 2006, and it was difficult to estimate the accurate exposure duration of MetS. Therefore, the exposure duration bias might misestimate the effects of MetS-recovery and MetS-stable groups. Fourth, the treatment rates of hypertension, diabetes and hyperlipidemia were low, which might lead to the high incidence rate of CVD. However, we have adjusted for the use of anti-hypertensive, anti-diabetic, and lipid-lowering drugs in the multivariate analysis, and still found the significant effects of MetS development and MetS recovery. Therefore, the potential bias of the low treatment rates was small. Finally, although we have adjusted for multiple confounding factors, other unmeasured variables such as genetic susceptibility, might exist and these would influence our results.

In conclusion, dynamic changes of MetS altered the risks of CVD and all-cause mortality, especially in individuals with an early onset age. Our findings highlight the importance of dynamic changes of MetS and onset age on the prevention and control for CVD.

## Data Availability Statement

The raw data supporting the conclusions of this article will be made available by the authors, without undue reservation.

## Ethics Statement

The studies involving human participants were reviewed and approved by the ethics committee of Zhejiang University, School of Medicine, and Kailuan General Hospital. The patients/participants provided their written informed consent to participate in this study.

## Author Contributions

YZhu and SW designed the research. SW and SC collected the data. DH, XZ, CD, QW, YZhou, and ZJ analyzed the data. DH, YZhu, and XZ wrote the paper. All authors read and approved the final manuscript.

## Conflict of Interest

The authors declare that the research was conducted in the absence of any commercial or financial relationships that could be construed as a potential conflict of interest.

## Publisher's Note

All claims expressed in this article are solely those of the authors and do not necessarily represent those of their affiliated organizations, or those of the publisher, the editors and the reviewers. Any product that may be evaluated in this article, or claim that may be made by its manufacturer, is not guaranteed or endorsed by the publisher.

## Abbreviations

BMI: body mass index
CI: confidence interval
CVD: cardiovascular diseases
DBP: diastolic blood pressure
FBG: fasting blood glucose
HDL-C: high-density lipoprotein cholesterol
HR: hazard ratio
LDL-C: low-density lipoprotein cholesterol
MetS: metabolic syndrome
MI: myocardial infarction
SBP: systolic blood pressure
SD: standard deviation
TC: total cholesterol
TG: triglyceride
WC: waist circumference.
