# Supplementary material for: Dynamic Changes of Metabolic Syndrome Alter the Risks of Cardiovascular Diseases and All-Cause Mortality: Evidence From a Prospective Cohort Study
Source: Front Cardiovasc Med. 2021 Aug 4;8:706999. doi: 10.3389/fcvm.2021.706999 (PMC8371451; doi:10.3389/fcvm.2021.706999)
Supplement: Supplementary file 1 [file Data_Sheet_1.docx]

**Supplemental Table 1.** Characteristics of subjects by the dynamic patterns of MetS in 2006

|  | MetS-free | MetS-developed | MetS-recovery | MetS-stable | *P* value |
| --- | --- | --- | --- | --- | --- |
| Number | 19373 | 3277 | 2652 | 6179 |  |
| Mean age (SD), year | 46.8(12.1) | 49.7(11.1) | 50.0(11.2) | 51.9(10.3) | <0.001 |
| Female, n (%) | 4733(24.4) | 627(19.1) | 483(18.2) | 1291(20.9) | <0.001 |
| Mean BMI (SD), kg/m2 | 23.5(3.1) | 26.3(3.1) | 25.8(3.1) | 27.6(3.2) | <0.001 |
| High school or above, n (%) | 4230(21.8) | 580(17.7) | 414(15.6) | 1004(16.3) | <0.001 |
| Married, n (%) | 18514(95.6) | 3163(96.5) | 2579(97.3) | 6003(97.2) | <0.001 |
| Ever smokers, n (%) | 7361(38.0) | 1386(42.3) | 953(35.9) | 2505(40.5) | <0.001 |
| Ever drinkers, n (%) | 7571(39.1) | 1479(45.1) | 975(36.8) | 2626(42.5) | <0.001 |
| Inactive physical activity, n (%) | 1856(9.6) | 331(10.1) | 201(7.6) | 587(9.5) | 0.005 |
| High salt intake, n (%) | 1808(9.3) | 368(11.2) | 228(8.6) | 732(11.9) | <0.001 |
| Family history of MI, n (%) | 368(1.90) | 74(2.26) | 45(1.70) | 162(2.62) | 0.002 |
| Family history of stroke, n (%) | 834(4.30) | 156(4.76) | 102(3.85) | 329(5.32) | 0.002 |
| Mean SBP (SD), mmHg | 121.6(17.5) | 129.8(18.8) | 136.4(17.4) | 142.2(18.7) | <0.001 |
| Mean DBP (SD), mmHg | 79.1(10.2) | 83.5(10.9) | 87.1(10.3) | 90.2(11.2) | <0.001 |
| Mean WC (SD), cm | 82.4(9.5) | 89.1(9.4) | 90.2(7.9) | 93.4(8.2) | <0.001 |
| Mean FBG (SD), mmol/L | 4.97(0.90) | 5.41(1.43) | 5.61(1.61) | 6.58(2.30) | <0.001 |
| Mean TG (SD), mmol/L | 1.18(0.82) | 1.77(1.36) | 2.30(1.52) | 2.86(1.99) | <0.001 |
| Mean HDL-C (SD), mmol/L | 1.59(0.38) | 1.54(0.37) | 1.51(0.45) | 1.48(0.43) | <0.001 |
| MetS components, n (%) |  |  |  |  | <0.001 |
| 0 | 4916(25.4) | 82(2.5) | 0(0) | 0(0) |  |
| 1 | 7990(41.2) | 771(23.5) | 0(0) | 0(0) |  |
| 2 | 6467(33.4) | 2424(74.0) | 0(0) | 0(0) |  |
| 3 | 0(0) | 0(0) | 2320(87.5) | 3640(58.9) |  |
| 4 | 0(0) | 0(0) | 320(12.1) | 2243(36.3) |  |
| 5 | 0(0) | 0(0) | 12(0.4) | 296(4.8) |  |
| Elevated WC, n (%) ^a^ | 8251(42.6) | 2256(68.8) | 2413(91.0) | 5857(94.8) | <0.001 |
| Hypertension, n (%) ^b^ | 4588(23.7) | 1261(38.5) | 1658(62.5) | 4493(72.7) | <0.001 |
| Diabetes, n (%) ^c^ | 350(1.8) | 229(7.0) | 234(8.8) | 1670(27.0) | <0.001 |
| Hyperlipidemia, n (%) | 373(1.9) | 170(5.2) | 117(4.4) | 709(11.5) | <0.001 |
| Anti-hypertensive drug use, n (%) | 602(3.11) | 304(9.28) | 246(9.28) | 1293(20.93) | <0.001 |
| Anti-diabetic drug use, n (%) | 59(0.30) | 50(1.53) | 40(1.51) | 394(6.38) | <0.001 |
| Lipid-lowering drug use, n (%) | 5(0.03) | 1(0.03) | 7(0.26) | 172(2.78) | <0.001 |

^a^ Elevated WC was determined by the measured WC ≥85 cm for men or ≥80 cm for women.

^b^ Hypertension was determined by the measured blood pressure ≥140/90 mmHg or use of the anti-hypertensive drug.

^c^ Diabetes was determined by the measured FBG ≥7.0 mmol/L or use of the anti-diabetic drug.

MetS, metabolic syndrome; BMI, body mass index; MI, myocardial infarction; SBP, systolic blood pressure; DBP, diastolic blood pressure; WC, waist circumference; FBG, fasting blood glucose; TG, triglyceride; HDL-C, high-density lipoprotein cholesterol; SD, standard deviation

**Supplemental Table 2.** Characteristics of subjects by the dynamic patterns of MetS in 2008

|  | MetS-free | MetS-developed | MetS-recovery | MetS-stable | *P* value |
| --- | --- | --- | --- | --- | --- |
| Number | 19373 | 3277 | 2652 | 6179 |  |
| Mean age (SD), year | 48.9(12.2) | 51.7(11.2) | 52.0(11.4) | 54.0(10.5) | <0.001 |
| Female, n (%) | 4733(24.4) | 627(19.1) | 483(18.2) | 1291(20.9) | <0.001 |
| Mean BMI (SD), kg/m2 | 23.4(3.1) | 26.5(3.1) | 25.1(3.1) | 27.4(3.2) | <0.001 |
| High school or above, n (%) | 5428(28.0) | 696(21.2) | 552(20.8) | 1161(18.8) | <0.001 |
| Married, n (%) | 19182(99.0) | 3253(99.3) | 2640(99.6) | 6166(99.8) | <0.001 |
| Ever smokers, n (%) | 8669(44.8) | 1581(48.3) | 1260(47.5) | 2836(45.9) | <0.001 |
| Ever drinkers, n (%) | 8717(45.0) | 1699(51.9) | 1217(45.9) | 2931(47.4) | <0.001 |
| Inactive physical activity, n (%) | 4067(21.0) | 727(22.2) | 475(17.9) | 1244(20.1) | <0.001 |
| High salt intake, n (%) | 2111(10.9) | 457(14.0) | 260(9.8) | 820(13.3) | <0.001 |
| Family history of MI, n (%) | 368(1.90) | 74(2.26) | 45(1.70) | 162(2.62) | 0.002 |
| Family history of stroke, n (%) | 834(4.30) | 156(4.76) | 102(3.85) | 329(5.32) | 0.002 |
| Mean SBP (SD), mmHg | 122.1(17.5) | 138.9(17.8) | 130.8(18.5) | 143.8(18.8) | <0.001 |
| Mean DBP (SD), mmHg | 80.0(10.0) | 89.7(10.6) | 84.6(10.7) | 91.3(11.5) | <0.001 |
| Mean WC (SD), cm | 82.9(9.1) | 92.7(8.5) | 87.0(9.3) | 94.2(9.3) | <0.001 |
| Mean FBG (SD), mmol/L | 5.13(0.99) | 6.05(1.95) | 5.39(1.74) | 6.86(2.71) | <0.001 |
| Mean TG (SD), mmol/L | 1.14(0.76) | 2.40(2.75) | 1.43(1.00) | 2.82(2.45) | <0.001 |
| Mean HDL-C (SD), mmol/L | 1.55(0.43) | 1.44(0.55) | 1.51(0.94) | 1.41(0.51) | <0.001 |
| MetS components, n (%) |  |  |  |  | <0.001 |
| 0 | 4678(24.1) | 0(0) | 198(7.5) | 0(0) |  |
| 1 | 7991(41.3) | 0(0) | 846(31.9) | 0(0) |  |
| 2 | 6704(34.6) | 0(0) | 1608(60.6) | 0(0) |  |
| 3 | 0(0) | 2425(74.0) | 0(0) | 3352(54.2) |  |
| 4 | 0(0) | 784(23.9) | 0(0) | 2360(38.2) |  |
| 5 | 0(0) | 68(2.1) | 0(0) | 467(7.6) |  |
| Elevated WC, n (%) ^a^ | 8497(43.9) | 3075(93.8) | 1592(60.0) | 5854(94.7) | <0.001 |
| Hypertension, n (%) ^b^ | 4753(24.5) | 2229(68.0) | 1198(45.2) | 4768(77.2) | <0.001 |
| Diabetes, n (%) ^c^ | 414(2.1) | 476(14.5) | 179(6.8) | 2120(34.3) | <0.001 |
| Hyperlipidemia, n (%) | 509(2.6) | 269(8.2) | 135(5.1) | 940(15.2) | <0.001 |
| Anti-hypertensive drug use, n (%) | 603(3.11) | 359(10.96) | 227(8.56) | 1310(21.2) | <0.001 |
| Anti-diabetic drug use, n (%) | 102(0.53) | 117(3.57) | 52(1.96) | 716(11.59) | <0.001 |

^a^ Elevated WC was determined by the measured WC ≥85 cm for men or ≥80 cm for women.

^b^ Hypertension was determined by the measured blood pressure ≥140/90 mmHg or use of the anti-hypertensive drug.

^c^ Diabetes was determined by the measured FBG ≥7.0 mmol/L or use of the anti-diabetic drug.

MetS, metabolic syndrome; BMI, body mass index; MI, myocardial infarction; SBP, systolic blood pressure; DBP, diastolic blood pressure; WC, waist circumference; FBG, fasting blood glucose; TG, triglyceride; HDL-C, high-density lipoprotein cholesterol; SD, standard deviation

**Supplemental Table 3.** Hazard ratios and 95%CIs of the cardiovascular disease and all-cause mortality in MetS-developed and MetS-recovery groups according to the age of MetS status change after excluding incident events during the first two years of follow-up

| Outcomes | Hazard ratio (95% CI) † | | | *P* _interaction_ |
| --- | --- | --- | --- | --- |
|  | All age | <50 years | ≥50 years |  |
| MetS-developed vs MetS-free | |  |  |  |
| Cardiovascular disease |  |  |  |  |
| MetS-free | 1(reference) | 1(reference) | 1(reference) | 0.002 |
| MetS-developed | 1.79(1.48-2.16) | 2.31(1.61-3.32) | 1.62(1.30-2.02) |  |
| All-cause mortality |  |  |  |  |
| MetS-free | 1(reference) | 1(reference) | 1(reference) | 0.004 |
| MetS-developed | 1.05(0.88-1.25) | 1.54(1.05-2.26) | 0.96(0.79-1.17) |  |
| MetS-recovery vs MetS-stable | |  |  |  |
| Cardiovascular disease |  |  |  |  |
| MetS-stable | 1(reference) | 1(reference) | 1(reference) | 0.011 |
| MetS-recovery | 0.63(0.51-0.79) | 0.43(0.26-0.70) | 0.72(0.56-0.93) |  |
| All-cause mortality |  |  |  |  |
| MetS-stable | 1(reference) | 1(reference) | 1(reference) | 0.096 |
| MetS-recovery | 0.88(0.73-1.07) | 0.66(0.40-1.08) | 0.93(0.76-1.14) |  |

Cardiovascular disease was the composite of myocardial infarction, stroke, and heart failure.

† Hazard ration was adjusted for age, sex, marital status, education level, smoking status, drinking status, physical activity level, salt intake, use of anti-hypertensive drug, anti-diabetic drug, lipid-lowering drug, and family history of myocardial infarction, stroke.

MetS, metabolic syndrome; CI, confidence interval

**Supplemental Table 4.** Hazard ratios and 95%CIs of the subtypes of cardiovascular disease in the MetS-developed group according to the age of MetS development after excluding incident events during the first two years of follow-up

| Outcomes | Hazard ratio (95% CI) † | | | *P* _interaction_ |
| --- | --- | --- | --- | --- |
|  | All age | <50 years | ≥50 years |  |
| Myocardial infarction |  |  |  |  |
| MetS-free | 1(reference) | 1(reference) | 1(reference) | 0.062 |
| MetS-developed | 1.49(0.92-2.42) | 2.66(1.09-6.50) | 1.20(0.67-2.15) |  |
| Stroke |  |  |  |  |
| MetS-free | 1(reference) | 1(reference) | 1(reference) | 0.024 |
| MetS-developed | 1.71(1.36-2.14) | 2.08(1.35-3.19) | 1.57(1.20-2.04) |  |
| Ischemic stroke |  |  |  |  |
| MetS-free | 1(reference) | 1(reference) | 1(reference) | 0.050 |
| MetS-developed | 1.73(1.37-2.19) | 1.98(1.24-3.17) | 1.63(1.24-2.14) |  |
| Hemorrhagic stroke |  |  |  |  |
| MetS-free | 1(reference) | 1(reference) | 1(reference) | 0.324 |
| MetS-developed | 1.23(0.64-2.37) | 1.76(0.62-5.00) | 0.99(0.42-2.33) |  |
| Heart failure |  |  |  |  |
| MetS-free | 1(reference) | 1(reference) | 1(reference) | 0.062 |
| MetS-developed | 1.98(1.24-3.15) | 3.52(1.25-9.92) | 1.77(1.04-2.99) |  |

Stroke was the composite of ischemic stroke and hemorrhagic stroke.

† Hazard ration was adjusted for age, sex, marital status, education level, smoking status, drinking status, physical activity level, salt intake, use of anti-hypertensive drug, anti-diabetic drug, lipid-lowering drug, and family history of myocardial infarction, stroke.

MetS, metabolic syndrome; CI, confidence interval

**Supplemental Table 5.** Hazard ratios and 95%CIs of the subtypes of cardiovascular disease in the MetS- recovery group according to the age of MetS recovery after excluding incident events during the first two years of follow-up

| Outcomes | Hazard ratio (95% CI) † | | | *P* _interaction_ |
| --- | --- | --- | --- | --- |
|  | All age | <50 years | ≥50 years |  |
| Myocardial infarction |  |  |  |  |
| MetS-stable | 1(reference) | 1(reference) | 1(reference) | 0.283 |
| MetS-recovery | 0.75(0.46-1.24) | 0.54(0.20-1.47) | 0.90(0.50-1.61) |  |
| Stroke |  |  |  |  |
| MetS-stable | 1(reference) | 1(reference) | 1(reference) | 0.035 |
| MetS-recovery | 0.61(0.46-0.79) | 0.41(0.23-0.74) | 0.69(0.51-0.93) |  |
| Ischemic stroke |  |  |  |  |
| MetS-stable | 1(reference) | 1(reference) | 1(reference) | 0.056 |
| MetS-recovery | 0.59(0.45-0.78) | 0.42(0.23-0.77) | 0.66(0.48-0.91) |  |
| Hemorrhagic stroke |  |  |  |  |
| MetS-stable | 1(reference) | 1(reference) | 1(reference) | 0.267 |
| MetS-recovery | 0.73(0.36-1.47) | 0.34(0.04-2.85) | 0.86(0.41-1.83) |  |
| Heart failure |  |  |  |  |
| MetS-stable | 1(reference) | 1(reference) | 1(reference) | 0.172 |
| MetS-recovery | 0.61(0.33-1.10) | 0.30(0.07-1.37) | 0.72(0.37-1.39) |  |

Stroke was the composite of ischemic stroke and hemorrhagic stroke.

† Hazard ration was adjusted for age, sex, marital status, education level, smoking status, drinking status, physical activity level, salt intake, use of anti-hypertensive drug, anti-diabetic drug, lipid-lowering drug, and family history of myocardial infarction, stroke.

MetS, metabolic syndrome; CI, confidence interval

**Supplemental Table 6.** Hazard ratios and 95%CIs of cardiovascular disease for all variables in the multivariable Cox regression model

| Variables | Hazard ratio (95% CI) | *P* value |
| --- | --- | --- |
| MetS-free | 1(reference) |  |
| MetS-developed | 1.92(1.64-2.25) | < 0.001 |
| MetS-recovery | 1.42(1.17-1.72) | < 0.001 |
| MetS-stable | 2.31(2.03-2.63) | < 0.001 |
| Age | 1.052(1.047-1.058) | < 0.001 |
| Sex (male) | 1(reference) |  |
| Sex (female) | 0.54(0.46-0.64) | < 0.001 |
| The married | 1(reference) |  |
| The unmarried | 1.20(0.75-1.91) | 0.442 |
| Primary school or below | 1(reference) |  |
| Middle school | 0.99(0.84-1.16) | 0.884 |
| High school or above | 0.61(0.49-0.76) | < 0.001 |
| Never smokers | 1(reference) |  |
| Ever smokers | 1.33(1.17-1.51) | < 0.001 |
| Never drinkers | 1(reference) |  |
| Ever drinkers | 0.86(0.75-0.98) | 0.021 |
| Active physical activity | 1(reference) |  |
| Inactive physical activity | 1.05(0.94-1.18) | 0.376 |
| Low salt intake | 1(reference) |  |
| High salt intake | 1.02(0.86-1.21) | 0.805 |
| No family history of MI | 1(reference) |  |
| Family history of MI | 1.07(0.75-1.52) | 0.722 |
| No family history of stroke | 1(reference) |  |
| Family history of stroke | 1.07(0.83-1.36) | 0.607 |
| No use of anti-hypertensive drug | 1(reference) |  |
| Use of anti-hypertensive drug | 0.71(0.61-0.81) | < 0.001 |
| No use of anti-diabetic drug | 1(reference) |  |
| Use of anti-diabetic drug | 0.84(0.68-1.03) | 0.100 |
| No use of lipid-lowering drug | 1(reference) |  |
| Use of lipid-lowering drug | 0.80(0.50-1.26) | 0.331 |

Cardiovascular disease was the composite of myocardial infarction, stroke, and heart failure.

The multivariable Cox regression model included the dynamic patterns of MetS, age, sex, marital status, education level, smoking status, drinking status, physical activity level, salt intake, use of anti-hypertensive drug, anti-diabetic drug, lipid-lowering drug, and family history of myocardial infarction, stroke.

MetS, metabolic syndrome; CI, confidence interval; MI, myocardial infarction

**Supplemental Table 7.** Hazard ratios and 95%CIs of all-cause mortality for all variables in the multivariable Cox regression model

| Variables | Hazard ratio (95% CI) | *P* value |
| --- | --- | --- |
| MetS-free | 1(reference) |  |
| MetS-developed | 1.04(0.89-1.22) | 0.599 |
| MetS-recovery | 1.33(1.14-1.55) | < 0.001 |
| MetS-stable | 1.40(1.25-1.57) | < 0.001 |
| Age | 1.092(1.087-1.097) | < 0.001 |
| Sex (male) | 1(reference) |  |
| Sex (female) | 0.42(0.36-0.50) | < 0.001 |
| The married | 1(reference) |  |
| The unmarried | 1.33(0.91-1.93) | 0.138 |
| Primary school or below | 1(reference) |  |
| Middle school | 1.00(0.88-1.15) | 0.951 |
| High school or above | 0.69(0.57-0.83) | < 0.001 |
| Never smokers | 1(reference) |  |
| Ever smokers | 1.15(1.03-1.29) | 0.015 |
| Never drinkers | 1(reference) |  |
| Ever drinkers | 0.84(0.75-0.95) | 0.004 |
| Active physical activity | 1(reference) |  |
| Inactive physical activity | 1.02(0.92-1.13) | 0.698 |
| Low salt intake | 1(reference) |  |
| High salt intake | 1.06(0.91-1.24) | 0.436 |
| No family history of MI | 1(reference) |  |
| Family history of MI | 0.90(0.62-1.31) | 0.585 |
| No family history of stroke | 1(reference) |  |
| Family history of stroke | 0.80(0.62-1.03) | 0.084 |
| No use of anti-hypertensive drug | 1(reference) |  |
| Use of anti-hypertensive drug | 0.81(0.72-0.92) | 0.002 |
| No use of anti-diabetic drug | 1(reference) |  |
| Use of anti-diabetic drug | 0.76(0.63-0.91) | 0.004 |
| No use of lipid-lowering drug | 1(reference) |  |
| Use of lipid-lowering drug | 0.82(0.52-1.31) | 0.411 |

The multivariable Cox regression model included the dynamic patterns of MetS, age, sex, marital status, education level, smoking status, drinking status, physical activity level, salt intake, use of anti-hypertensive drug, anti-diabetic drug, lipid-lowering drug, and family history of myocardial infarction, stroke.

MetS, metabolic syndrome; CI, confidence interval; MI, myocardial infarction

**Supplemental Figure legends**

**Supplemental Figure 1.** Graphical description of the study design

This study included the exposure assessment window and follow-up window. The exposure assessment window was to define the dynamic patterns of MetS using data of the first three health examinations. The 2nd health examination (2008) was used to identify the occurrence of MetS status change, and the 3rd health examination (2010) was used to confirm the stable change of MetS status. The follow-up for long-term outcomes was initiated after the 3rd health examination (2010). The censoring dates were December 31, 2017 for cardiovascular disease and December 31, 2019 for all-cause mortality.

MetS, metabolic syndrome; “+”, MetS-present status; “-”, MetS-free status

**Supplemental Figure 2.** Examples of the dynamic patterns of MetS for exclusion

This figure showed several dynamic patterns of MetS for exclusion: (1) subjects with unstable change of MetS status; (2) subjects with change of MetS status at the third health examination. This study aimed to identify the stable change of MetS status. Therefore, we excluded subjects with unstable change of MetS status and those who could not assure the stable change of MetS status.

MetS, metabolic syndrome; “+”, MetS-present status; “-”, MetS-free status

**Supplemental Figure 3.** Incidence rates and risks of cardiovascular disease and all-cause mortality by the dynamic patterns of MetS after excluding incident events during the first two years of follow-up

Cardiovascular disease was the composite of myocardial infarction, stroke, and heart failure.

Hazard ration was adjusted for age, sex, marital status, education level, smoking status, drinking status, physical activity level, salt intake, use of anti-hypertensive drug, anti-diabetic drug, lipid-lowering drug, and family history of myocardial infarction, stroke.

MetS, metabolic syndrome; PY, person-year; CI, confidence interval

**Supplemental Figure 4.** Incidence rates and risks of the subtypes of cardiovascular disease by the dynamic patterns of MetS after excluding incident events during the first two years of follow-up

Stroke was the composite of ischemic stroke and hemorrhagic stroke.

Hazard ration was adjusted for age, sex, marital status, education level, smoking status, drinking status, physical activity level, salt intake, use of anti-hypertensive drug, anti-diabetic drug, lipid-lowering drug, and family history of myocardial infarction, stroke.

MetS, metabolic syndrome; PY, person-year; CI, confidence interval
