# Supplementary figures and images for: Dynamic Changes of Metabolic Syndrome Alter the Risks of Cardiovascular Diseases and All-Cause Mortality: Evidence From a Prospective Cohort Study
Source: Front Cardiovasc Med. 2021 Aug 4;8:706999. doi: 10.3389/fcvm.2021.706999 (PMC8371451; doi:10.3389/fcvm.2021.706999)

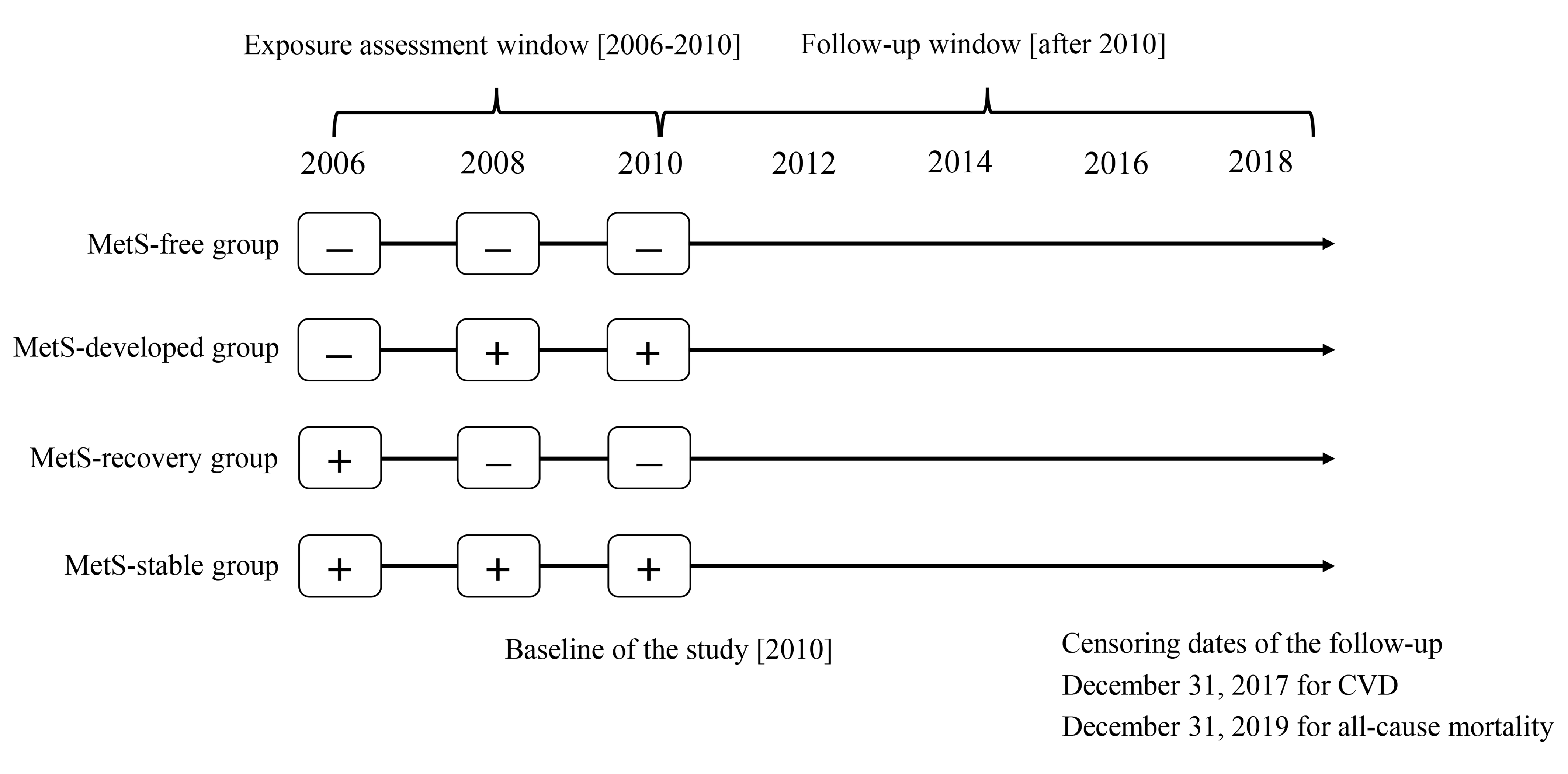

Supplement: Supplementary file 2 [file Image_1.TIF]

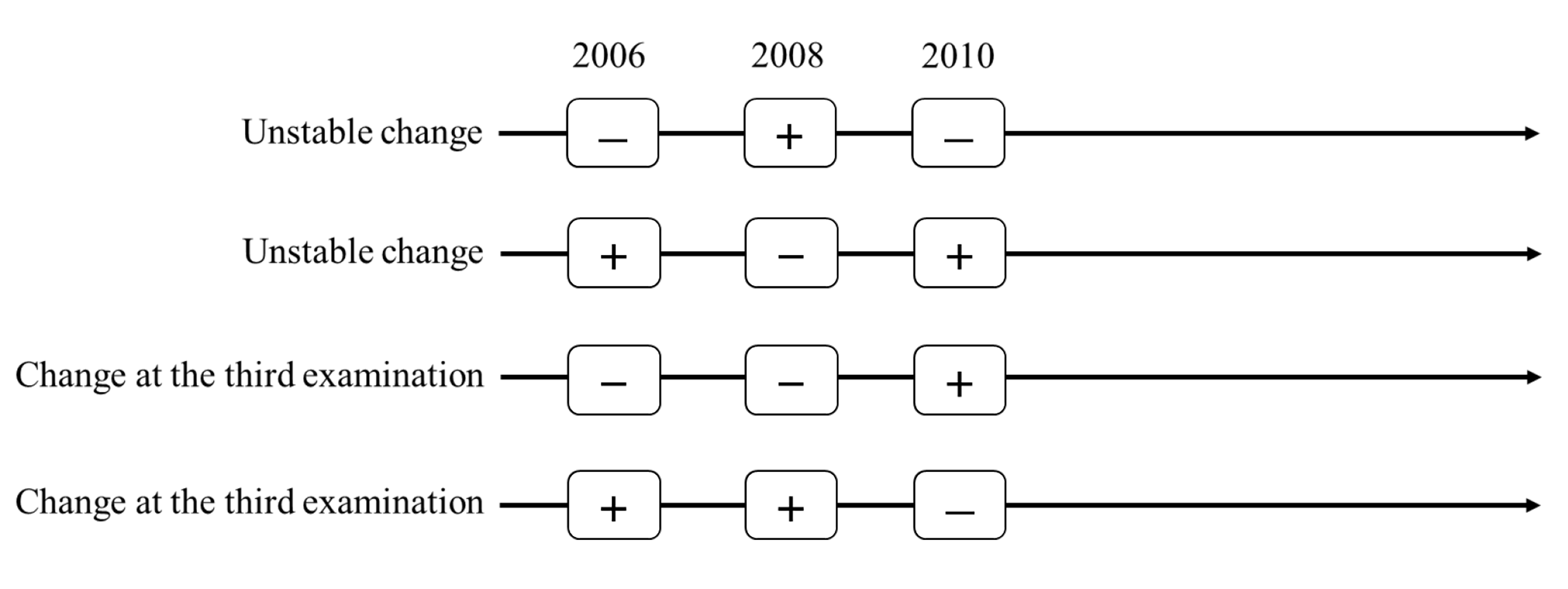

Supplement: Supplementary file 3 [file Image_2.TIF]

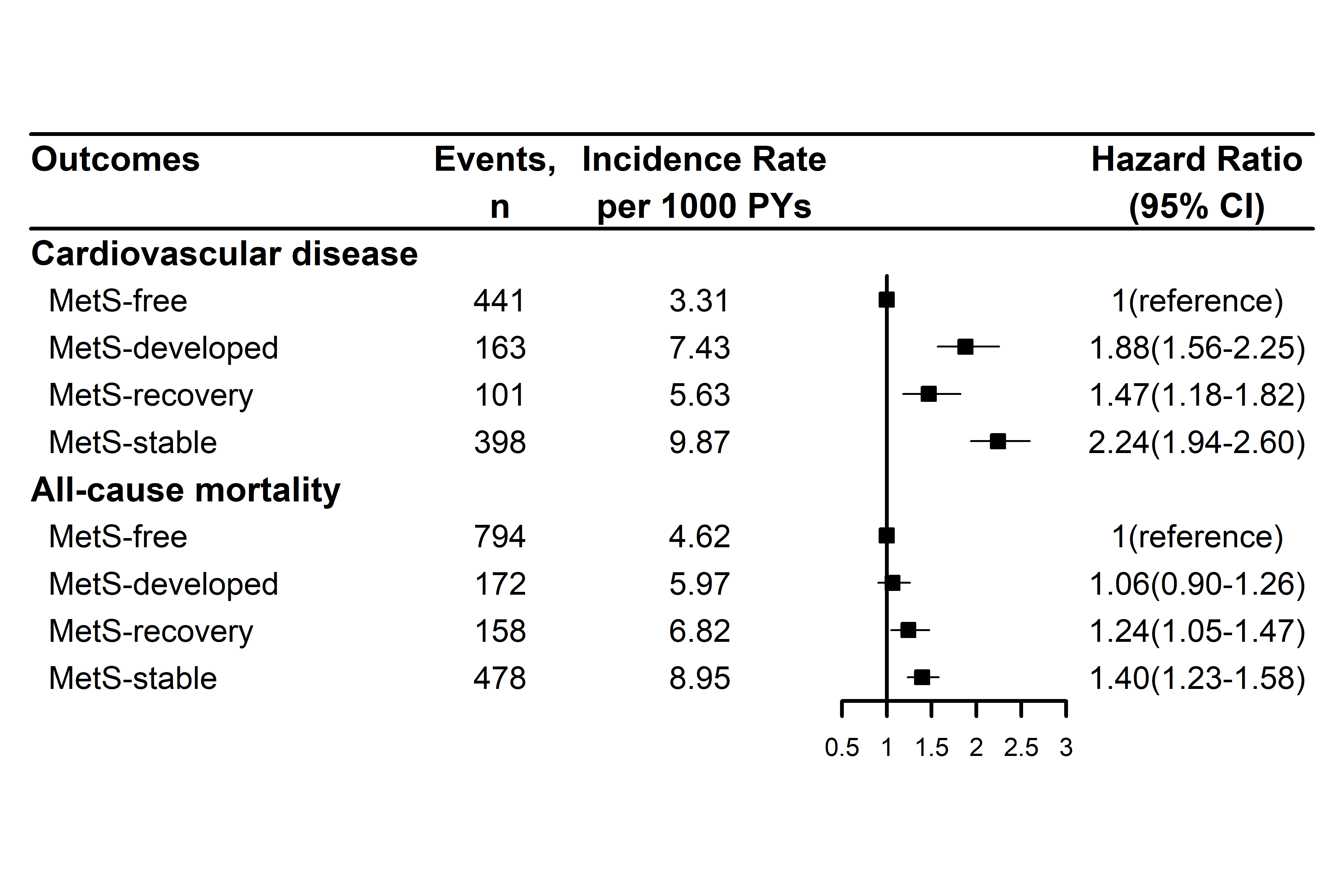

Supplement: Supplementary file 4 [file Image_3.TIF]

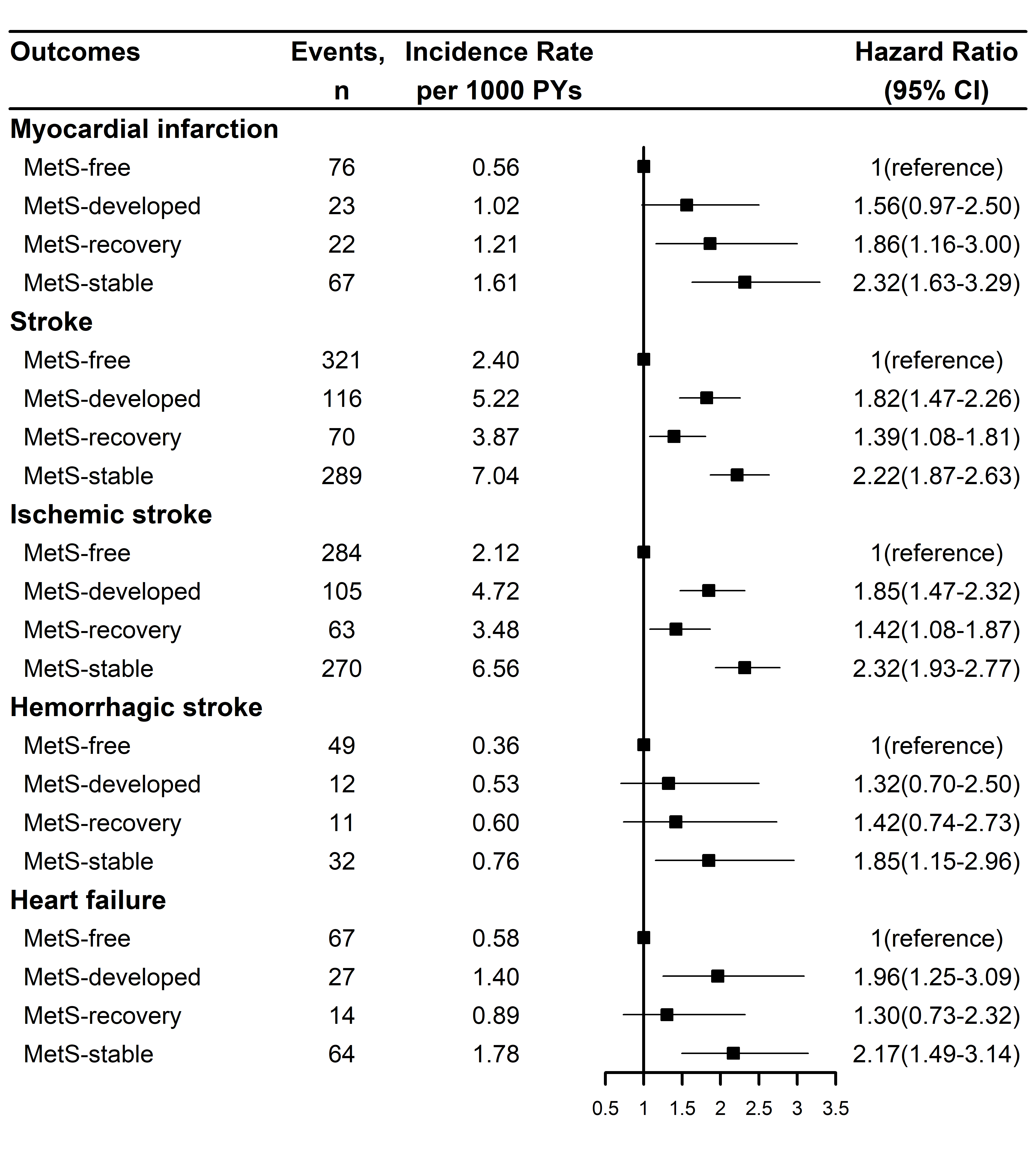

Supplement: Supplementary file 5 [file Image_4.TIF]
